# Development of BiFeO_3_‑Enhanced Cellulose Separators via Electrospinning for High-Performance Lithium-Ion Batteries

**DOI:** 10.1021/acsomega.6c02201

**Published:** 2026-07-13

**Authors:** Claudia C. Zuluaga-Gómez, Guillermo A. Narváez-Lozano, Sofia D. Robles-Alfonso, Junellie Cruz-Lebrón, Eduardo Nicolau

**Affiliations:** † Department of Chemistry, 19878 University of Puerto Rico, Rio Piedras Campus, P.O. Box 23346, San Juan, Puerto Rico 00931, United States; ‡ Department of Physics, University of Puerto Rico, San Juan, Puerto Rico 00925-2537, United States; § Molecular Sciences Research Center, University of Puerto Rico, San Juan, Puerto Rico 00926, United States

## Abstract

Lithium-ion battery (LIB) performance and safety are strongly dictated by the properties of the separator, which governs ion transport, interfacial stability, and resistance to dendrite penetration. In recent years, cellulose and its derivatives have emerged as a sustainable and high-performance alternative to commercial polypropylene separators for LIBs. Their polar, electrolyte-philic molecular structure improves wettability and ionic transport, promoting uniform Li-ion flux, and reduces the probability of dendrite formation. In this work, we developed a regenerated cellulose separator enhanced with ferroelectric BiFeO_3_ (BFO) nanoparticles via electrospinning followed by an alkaline hydrolysis process. The cellulose acetate precursor produced a highly porous and polar fibrous network that facilitated electrolyte uptake and ionic transport, while the ferroelectric properties of BFO contributed to internal electric field redistribution at the electrode–separator interface, mitigating dendrite nucleation and growth. This synergistic effect led to significant improvements in interfacial stability and ion transport. Structural, chemical, and morphological analyses (FT-IR, EDS, and SEM) confirmed successful regeneration and uniform nanoparticle incorporation. Electrochemical benchmarking against RE:C, RE:C–Bi_2_O_3_, and RE:Fe_2_O_3_ separators, as well as pristine regenerated cellulose fibers and commercial polypropylene separators, demonstrated a clear performance advantage for the RE:C-BFO separator. With an average thickness of 40 μm, this separator exhibited a 1.91 factor improvement in electrolyte wettability and nearly a 2 orders of magnitude enhancement in ionic conductivity. Electrochemical testing revealed a low charge transfer resistance of 48 Ω and excellent cycling stability, maintaining ∼78% capacity retention after 100 cycles and enabling stable operation for up to 500 cycles. The discharge capacity of the RE:C-BFO separator decreased from 326 mAh/g at a current density of 0.2 mA/cm^2^ to 252 mAh/g after 100 cycles at 0.5 mA/cm^2^, representing significantly better performance compared with the control separators and approaching the theoretical specific capacity of graphite (372 mAh/g). Overall, these results highlight the strong potential of oxide-enhanced regenerated cellulose (RE:C-MFO) separators to improve electrolyte wetting, enhance Li-ion conduction, and suppress dendrite nucleation through internal electric field homogenization, providing a promising pathway toward safer, more durable next-generation LIBs.

## Graphical abstract

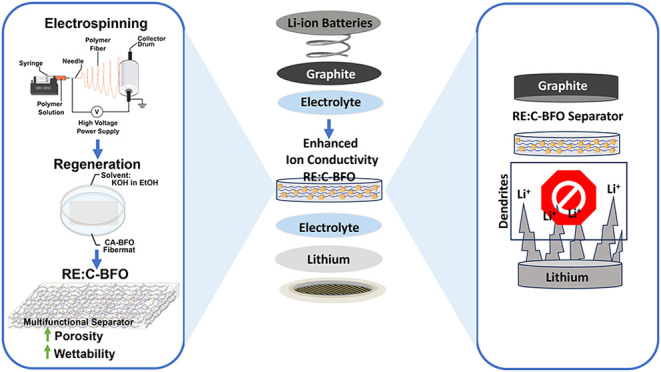

## 1 Introduction

The global demand for LIBs is projected to reach 4.7 terawatt-hours by 2030, driven by the growing needs in home energy storage, grid-scale systems, and electric vehicles. Despite their widespread use, LIBs face challenges related to rapid degradation, limited lifespan, and safety risks.^1,2^ The separator plays a critical role in preventing thermal runaway, suppressing dendrite penetration, and maintaining electrolyte retention. Since their commercialization in the early 1990s, LIBs have enabled modern portable electronics, electrified transport, and grid-scale storage due to their high energy density, long cycle life, and efficiency.^3–8^ The separator, despite being electrochemically inert, is a key component that physically isolates the electrodes while allowing lithium-ion transport. Its physicochemical characteristics, pore structure, and mechanical robustness strongly influence rate capability, cycling stability, and overall safety under normal and abusive conditions.^3–6,9–12^ One of the most critical limitations affecting LIBs is the formation and growth of lithium dendrites during repeated charge–discharge cycling. These metallic protrusions can penetrate the separator, leading to internal short circuits, capacity fading, and, in severe cases, thermal runaway.^4,13^ Commercial separators are commonly fabricated from polyolefins such as polypropylene and polyethylene, which offer good mechanical strength and low manufacturing costs. However, they remain limited by (i) poor thermal stability, (ii) insufficient resistance to dendrite penetration, and (iii) low electrolyte wettability, which restrict ion transport and interfacial stability.^5,10,11,14–17^ These limitations propel the development of new separators with improved wettability, enhanced thermal tolerance, and higher mechanical resistance. A variety of fabrication methods have been explored to optimize separator properties. Conventional commercial PP and PE separators are produced through dry stretching, wet stretching, or phase inversion, generating microporous membranes with controlled pore size.^18^ Although they are industrially robust, these techniques offer limited tunability of pore architecture and rely on hydrophobic polymers with inherently poor electrolyte affinity. Phase inversion and solution casting methods enable porous separators but often result in irregular pore distribution, modest mechanical strength, or insufficient thermal resistance for high-power and fast-charging applications.^19,20^ In contrast, electrospinning has emerged as one of the most versatile and powerful approaches for separator fabrication.^16,17^ This technique produces nonwoven nanofibrous mats with interconnected pores, tunable fiber diameter, high surface area, and controllable morphology, enabling superior electrolyte uptake, rapid Li^+^ diffusion pathways, and enhanced thermal stability compared to separators produced by conventional methods. Moreover, electrospinning allows uniform dispersion of functional inorganic nanoparticles such as metal oxides or ferroelectrics within polymeric fibers, producing multifunctional separators capable of modulating ion transport, redistributing internal electric fields, or suppressing dendrite propagation.^17,21,22^ Cellulose-based separators, including cellulose acetate (CA), regenerated cellulose, nanofibers, and nanocrystals, have emerged as sustainable alternatives to polyolefins due to their abundance, thermal stability, and intrinsic hydrophilicity, which translate into higher electrolyte uptake and improved ion conductivity.^20–22^ Recently, oxide fillers such as Al_2_O_3_, TiO_2_, and SiO_2_ have been incorporated into polymeric separators to enhance mechanical strength, wettability, and thermal resistance.^23,24^ Building upon this approach, ferroelectric oxides such as BFO have attracted increasing interest due to the ability to regulate local electric fields, homogenize Li^+^ distribution, and mitigate dendritic growth at the electrode–separator interface.^23–25^ In most studies, oxide-based nanoparticles have been incorporated into separators to improve electrolyte wettability, ion transport, and cycling stability. Table 1 summarizes representative results reported in the literature and compares them with the present work, where only the separator composition was modified while keeping the graphite anode constant, enabling a direct assessment of separator’s influence on LIB performance. In this work, we present the development of oxide-enhanced regenerated cellulose separators fabricated via electrospinning, incorporating Bi_2_O_3_, Fe_2_O_3_, and BiFeO_3_ as functional additives. These multifunctional oxide-modified separators, RE:C–Bi_2_O_3_, RE:C–Fe_2_O_3_, and RE:C-BFO, are collectively referred to as RE:C-MFO. The novelty of this approach lies in combining the intrinsic advantages of electrospun regenerated cellulose, including sustainability, high porosity, and strong electrolyte affinity, with the multifunctional characteristics of metal oxide nanoparticles to enhance mechanical robustness, improve electrolyte wettability, and suppress dendrite formation. This integrated strategy enables a systematic evaluation of how multifunctional oxides modulate separator performance and provides a promising pathway for the design of safer and high-performance next-generation LIBs.

**Table 1:** Comparison of Separator Properties and Electrochemical Performance of Cellulose-Based LIB Separators Using Graphite Anodes in Previous Studies and In This Work

ref	separator	fabrication method	thickness [μm]	porosity [%]	electrolyte uptake [%]	first specifics capacity [mAh/g]	capacity retention [%]	cyclability [cycles]
2014 ^26^	FCCN (flame-retardant cellulose composite)	papermaking + ceramic coating	25	∼70	∼210	350	70	300
anode: MCMB graphite+PVDF								
2018 ^27^	CA-TiO_2_	electrospinning	40	∼60	∼210	365	60	200
anode: MCMB graphite+PVDF								
2023 ^28^	electrospun cellulose nanofiber	electrospinning	35	∼78	∼240	310	80	500
anode: graphite+PVDF								
2025 ^29^	CA + cellulose nanocrystals	electrospinning	50	∼62	∼350	330	72	300
anode: graphite+PVDF								
2025	SC (Celgard)	commercial	25	∼88	∼200	346.11	52	500
this work	RE:C	electrospinning + regeneration	70	∼58	∼427	51.99		5
anode: graphite+CB+PVDF	RE:C–Bi_2_O_3_		50	∼59	∼433	360.75	37	500
	RE:C–Fe_2_O_3_		50	∼57	∼564	391.91	65	500
	RE:C-BFO		40	∼63	∼382	326.48	77	500

## 2 Materials and Methods

### 2.1 Materials

Cellulose acetate (CA, average Mn: ∼30,000), acetone (HPLC Plus, ≥99.9%), *N*,*N*-dimethylacetamide (DMAc, Reagent Plus, ≥99%), potassium hydroxide (KOH, analytical grade), ethanol (EtOH, ≥99.5%), graphite (powder, ∼200 mesh, 99.9999%), carbon black, poly­(vinylidene fluoride) (PVDF, average Mw: ∼534,000), *N*-methyl-2-pyrrolidone (NMP, ACS Reagent, ≥99%), and iron­(III) oxide (Fe_2_O_3_, 99.998%) were purchased from Sigma-Aldrich, and high-purity bismuth­(III) oxide (Bi_2_O_3_, 99.9%) was obtained from Fluka. The commercial separator used in this study was Celgard 2500 (monolayer polypropylene membrane, thickness ≈ 25 μm), purchased from Celgard. All chemicals were used as received without further purification. All chemical materials for separator fabrication and battery assembly were handled inside an Ar-filled glovebox with water (H_2_O) and oxygen (O_2_) contents maintained below 0.5 ppm.

### 2.2 Synthesis of the Ferroelectric Material

Bismuth ferrite (BiFeO_3_, BFO) powder was synthesized via a conventional solid-state reaction followed by thermal annealing. Briefly, stoichiometric amounts of Fe_2_O_3_ and Bi_2_O_3_ with a slight excess of Bi_2_O_3_ were homogenized by high-energy ball milling, dried, and subsequently calcined at high temperature to obtain phase-pure BFO. A detailed description of the synthesis and characterization of BFO has been reported previously in a previous article *“Holey Graphene/Ferroelectric/Sulfur Composite Cathodes for High-Capacity Lithium–Sulfur Batteries”* published in *ACS Omega*.^30^

### 2.3 Electrospinning

The separators were fabricated by preparing solutions in a solvent mixture of acetone and DMAc (2:1, v/v). The CA solution was prepared at room temperature by dissolving CA at a concentration of 17 wt % in the solvent mixture, followed by overnight stirring. In addition to the pristine CA separator, three composite separators were prepared following the same procedure by incorporation of 0.25 wt % of Bi_2_O_3_, Fe_2_O_3_, or BiFeO_3_. The oxide loading of 0.25 wt % was selected based on preliminary optimization studies and previous experimental experience in our laboratory. Earlier investigations using different materials and concentrations ranging from 0 to 5 wt % demonstrated that increasing oxide loading promotes nanoparticle agglomeration within the electrospun fibers, resulting in heterogeneous morphologies, disruption of the nanofibrous network, and deterioration of mechanical properties. In the present system, preliminary experiments with varying oxide concentrations confirmed that higher loading (>0.25 wt %) caused aggregation and significant distortion of the electrospun fibers, negatively affecting membrane uniformity, porosity, and ionic transport pathways. In contrast, lower loading (<0.25 wt %) resulted in insufficient oxide incorporation, limiting interfacial interactions and reducing the ferroelectric contribution required to effectively modulate Li^+^ flux. A loading of 0.25 wt % provided the most uniform fiber morphology, with well-dispersed oxide nanoparticles and preserved interconnected porosity. This loading represents an optimal balance between structural integrity and functional performance, ensuring enhanced electrochemical properties without compromising membrane stability, making it the most suitable composition for this study. In each case, the oxide powder was dispersed in the solvent mixture by sonication for 30 min, after which 17% CA was added, and the solution was stirred overnight. This process yielded the CA-Bi_2_O_3_, CA-Fe_2_O_3_, and CA-BFO composite separators, hereafter collectively referred to as CA-MFO (cellulose acetate multifunctional oxide) separators. The 17% CA separator and the commercial polypropylene separator were used as reference materials for comparison. All the solutions were prepared and electrospun using a γ high-voltage generator onto a round copper collector covered with aluminum foil. Each solution was loaded into a 5 mL plastic syringe equipped with a 23G stainless-steel needle and subjected to the electrospinning process. The nanofibers were deposited under an applied voltage of 12 kV to 15 kV, with a feeding rate of 0.50 mL/h and a fixed tip-to-collector distance of 15 cm. The separators obtained were detached from the collector and transferred to a desiccator. Regeneration of the CA-based membranes was performed in 0.05 M KOH aqueous ethanol solution at ambient temperature for 24 h, followed by rinsing with nanopure water and drying under vacuum at 80 °C for 72 h to obtain regenerated cellulose (RE:C) separators incorporating multifunctional oxides; three composite membranes were fabricated using Bi_2_O_3_ (RE:C–Bi_2_O_3_), Fe_2_O_3_ (RE:C–Fe_2_O_3_), and BiFeO_3_ (RE:C-BFO). For simplicity, these oxide-modified regenerated cellulose separators are hereafter referred to as RE:C-MFO (regenerated cellulose multifunctional oxide) separators. A schematic illustration of the composite separator preparation is shown in Scheme 1.

**Scheme 1:**
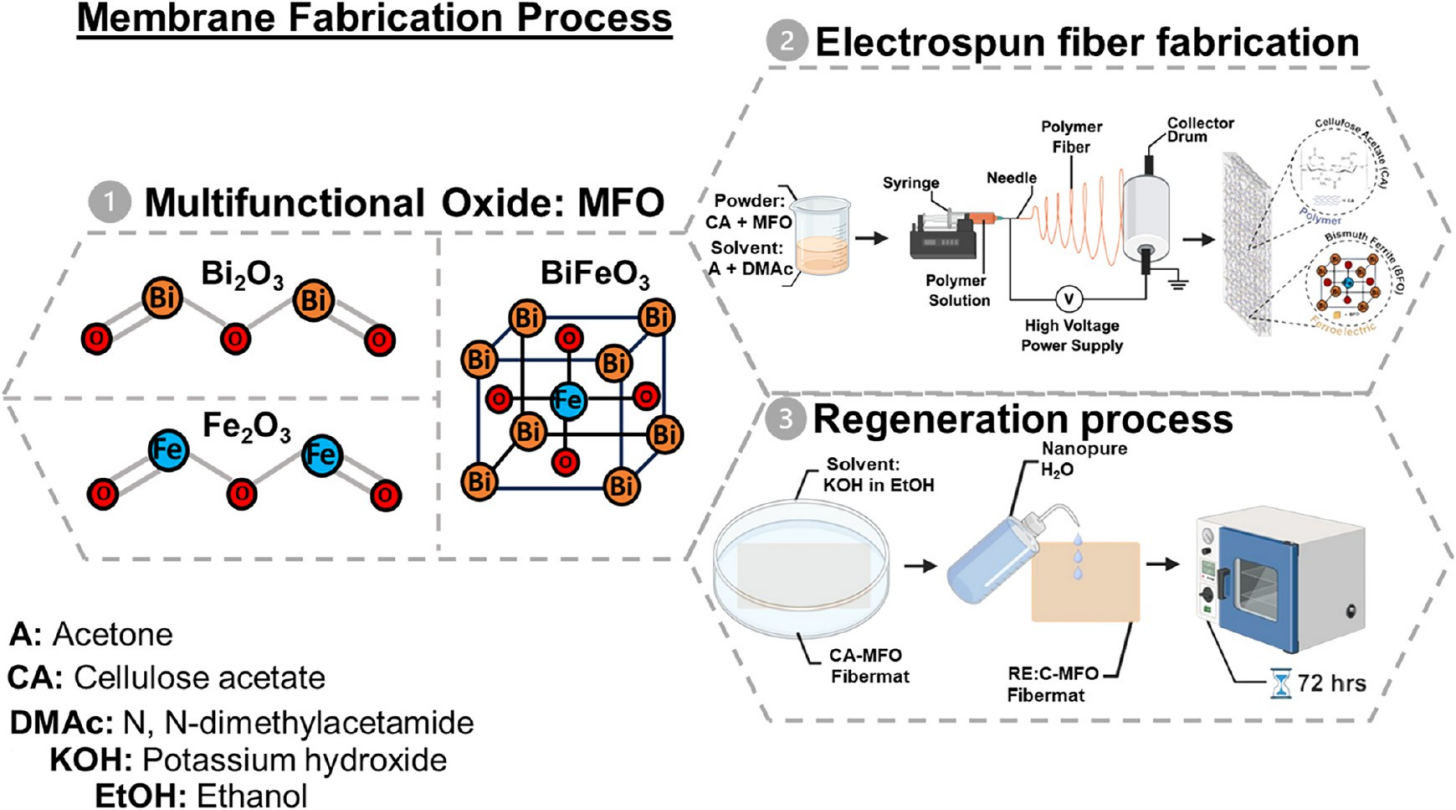
Schematic Illustration of the RE:C-MFO Fiber Preparation by Electrospinning and the Separator Regeneration Treatment

### 2.4 Graphite Anode Fabrication

The fabrication of the graphite anode involved the preparation of a slurry in which PVDF was employed as a binder to ensure adequate adhesion and mechanical stability. For this purpose, a PVDF solution was prepared by dissolving 5 wt % PVDF powder in NMP under continuous magnetic stirring at room temperature until a homogeneous mixture was obtained. The anode slurry was prepared by mixing 80 wt % graphite, 10 wt % carbon black, and 10 wt % PVDF:NMP solution. The mixture was manually homogenized in a mortar until a uniform slurry was formed, adding NMP if necessary to achieve the desired viscosity. The slurry was then coated onto copper foil using the doctor blade method. After coating, the electrodes were dried overnight at 80 °C in a vacuum oven to remove the residual NMP solvent and ensure electrode stability. The coated anodes were punched into round discs with a diameter of 10 mm and further dried at 60 °C under vacuum for ∼2 h before being transferred into an Ar-filled glovebox. A schematic diagram of the fabrication of the graphite anodes is shown in Scheme 2.

**Scheme 2:**
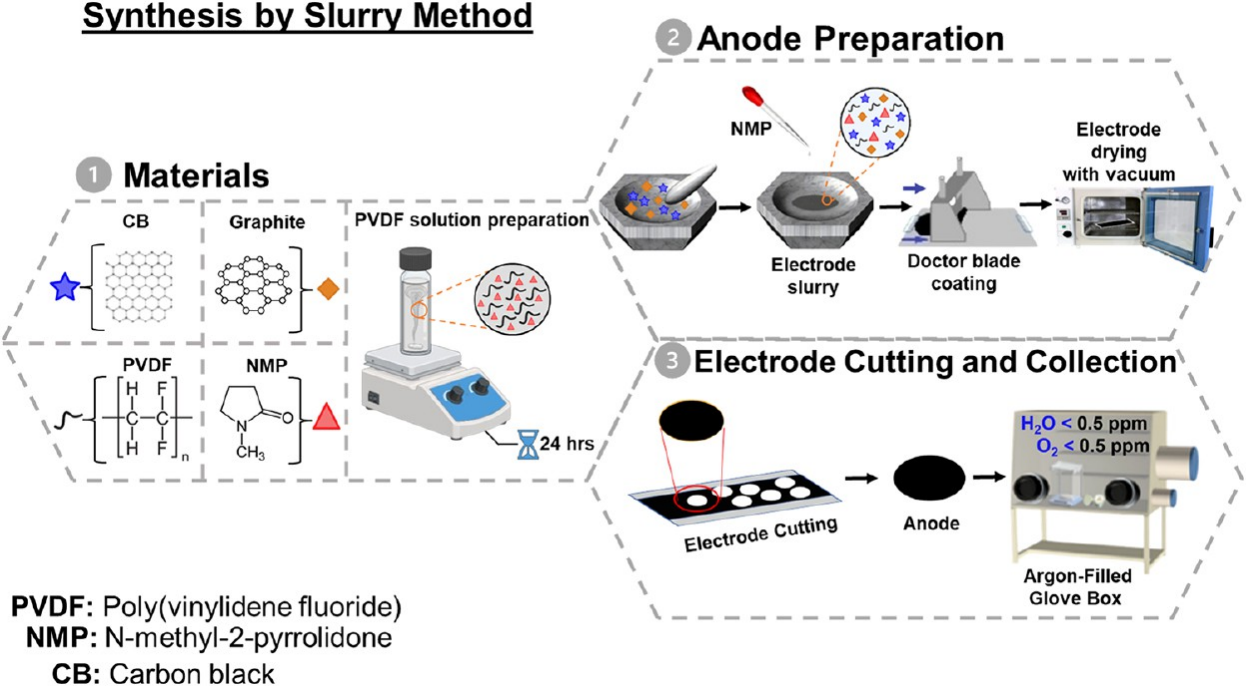
Schematic Diagram of the Graphite Anode in the Fabrication Process

### 2.5 Coin Cell Assembly

CR2032 coin-type half-cells were assembled in an Ar-filled glovebox with H_2_O and O_2_ contents maintained below 0.5 ppm. A lithium chip was used as a counter electrode, while the composite graphite anode composed of 80 wt % graphite, 10 wt % carbon black, and 10 wt % PVDF: NMP solution, with a diameter of 10 mm (MTI corporation), served as the working electrode. Separators included the pristine RE:C, RE:C-MFO (RE:C–Bi_2_O_3_, RE:C–Fe_2_O_3_, RE:C-BiFeO_3_), and the commercial separators (CSs), all with a diameter of 16 mm. The cells were then activated by adding the electrolyte 1 M LiPF_6_ dissolved in a mixture of ethylene carbonate (EC), dimethyl carbonate (DMC), and diethyl carbonate (DEC) (weight ratio: 1:1:1). To calculate the proper electrolyte volume, 40 μL was used for ∼3.8 mg of active mass, corresponding to an anode loading of 7 mAh/g.

### 2.6 Characterization Methods

The 17 wt % CA and commercial separators were characterized using Fourier transform infrared spectroscopy (FT-IR, Bruker α II Compact FT-IR ATR), energy dispersive X-ray spectroscopy (EDS), and scanning electron microscopy (SEM, Thermo Scientific Phenom Pharos G2) operated at acceleration voltages of 10 kV and 15 kV to examine surface morphology. Wettability was evaluated by contact angle measurements (Krüss Drop Angle Analyzer). The pore size distribution and porosity of the electrospun separators were determined using a capillary flow porometer (Quantachrome Instruments, Porometer 3Gzh). The mechanical properties were assessed through tensile strength testing (Brookfield CT3 Texture Analyzer). Each diaphragm was cut into a rectangle of 20 × 9 mm, and there were at least three parallel samples analyzed for each group. Then, the thickness of each sample was measured using a vernier caliper. Wettability between the separator and liquid electrolyte was further evaluated by measuring static contact angles with a video contact angle tester. Electrochemical performance was evaluated using CR2032 Li-ion half-cells. Galvanostatic charge–discharge tests were performed with an eight-channel battery analyzer (CD, Landt Battery Test System CT3002) at different current densities calculated according to the anode area (0.786 cm^2^), ranging from 0.2 to 0.5 mA cm^–2^. Electrochemical impedance spectroscopy (EIS) measurements were performed using an Arbin Instruments, MITS Pro 8.0 system at the open-circuit potential over a frequency range of 1 MHz to 0.01 Hz, with an AC amplitude of 10 mV and 10 points per decade. The ion conductivity (σ) was calculated according to the equation σ = *t*/(*R*_s_ × *A*), where *A* is the contact area of the polymer electrolyte separator (cm^2^), *t* is the separator thickness (cm), and *R*_*s*_ is the solution resistance obtained from the intercept of the real axis in the Nyquist impedance plot.

## 3 Results and Discussion

### 3.1 Structural and Morphological Characterization of BiFeO_3_

The X-ray diffraction pattern of the synthesized BiFeO_3_ powder measured at room temperature is shown in Figure 1a. The diffraction peaks are indexed to the rhombohedral perovskite structure of BFO with the space group *R*3*c* (JCPDS No. 86–1518), consistent with the findings of Heng and coautors.^31^ The most prominent reflections at 2θ ≈ 22.4, 31.9, 32.1, 39.5, 45.7, 51.1, and 57.1° correspond to the (012), (104), (110), (202), (024), (116), and (300) planes, respectively, in agreement with previous reports.^32–34^ A weak secondary peak around 2θ ≈ 28° can be attributed to traces of a pyrochlore-related phase (Bi_2_Fe_4_O_9_), commonly detected as a minor impurity in BFO prepared by a solid-state reaction.^35^ The average crystallite size, calculated from the (110) reflection using the Scherrer equation, was ∼53.6 nm, indicating the formation of nanocrystalline BFO with high phase purity and a well-defined perovskite structure. The ferroelectric properties of the BFO nanoparticles were further examined through the polarization–electric field (P-E) hysteresis loops, as shown in Figure 1b. The loop exhibits a characteristic slim but closed shape, confirming the intrinsic ferroelectric nature of the BFO phase. The remnant polarization (P_r_) was approximately 8.5 μCcm^–2^ at an applied electric field of 3 kV cm^–1^, consistent with previously reported values for bulk BFO ceramics obtained by solid-state synthesis.^32^ The slightly rounded loop profile suggests the presence of moderate leakage currents, typically associated with oxygen vacancies and mixed Fe^2+^/Fe^3+^ valence states within the perovskite lattice.^36^ BFO is a well-known multiferroic material exhibiting both ferroelectric and antiferromagnetic ordering at room temperature. Its spontaneous polarization generates an internal electric field capable of modulating interfacial charge transport and enhancing electrochemical performance when incorporated into polymer matrices or battery components.^37^ These structural and ferroelectric results confirm that the synthesized BFO retains its multifunctional properties, making it a promising electroactive additive for developing oxide-enhanced cellulose acetate separators in lithium-ion batteries. The surface morphology of the synthesized BFO powder was examined by SEM, as shown in Figure 1c. The micrograph reveals a quasi-spherical morphology with moderately agglomerated grains having sizes below 100 nm, which is typical of BFO powders obtained through solid-state synthesis, where particle sintering occurs due to the high surface energy and partial diffusion of ions during calcination. The dense and interconnected grain structure suggests effective crystallization and strong interparticle bonding, consistent with the formation of a well-defined perovskite phase observed in the XRD analysis. The elemental composition of the synthesized BFO powder was confirmed through energy dispersive X-ray spectroscopy, as shown in Figure 1d. Distinct peaks corresponding to Bi (Lα), Fe (Kα), and O (Kα) were observed, confirming the stoichiometric formation of BFO. The EDS elemental mapping images in Figure 1e–h show a uniform spatial distribution of Bi, Fe, and O throughout the analyzed region, validating the compositional homogeneity of the synthesized material. The uniform elemental dispersion further supports the successful formation of the BFO perovskite structure, which is crucial for maintaining stable ferroelectric and multiferroic behavior in subsequent electrochemical applications.

**Figure 1:**
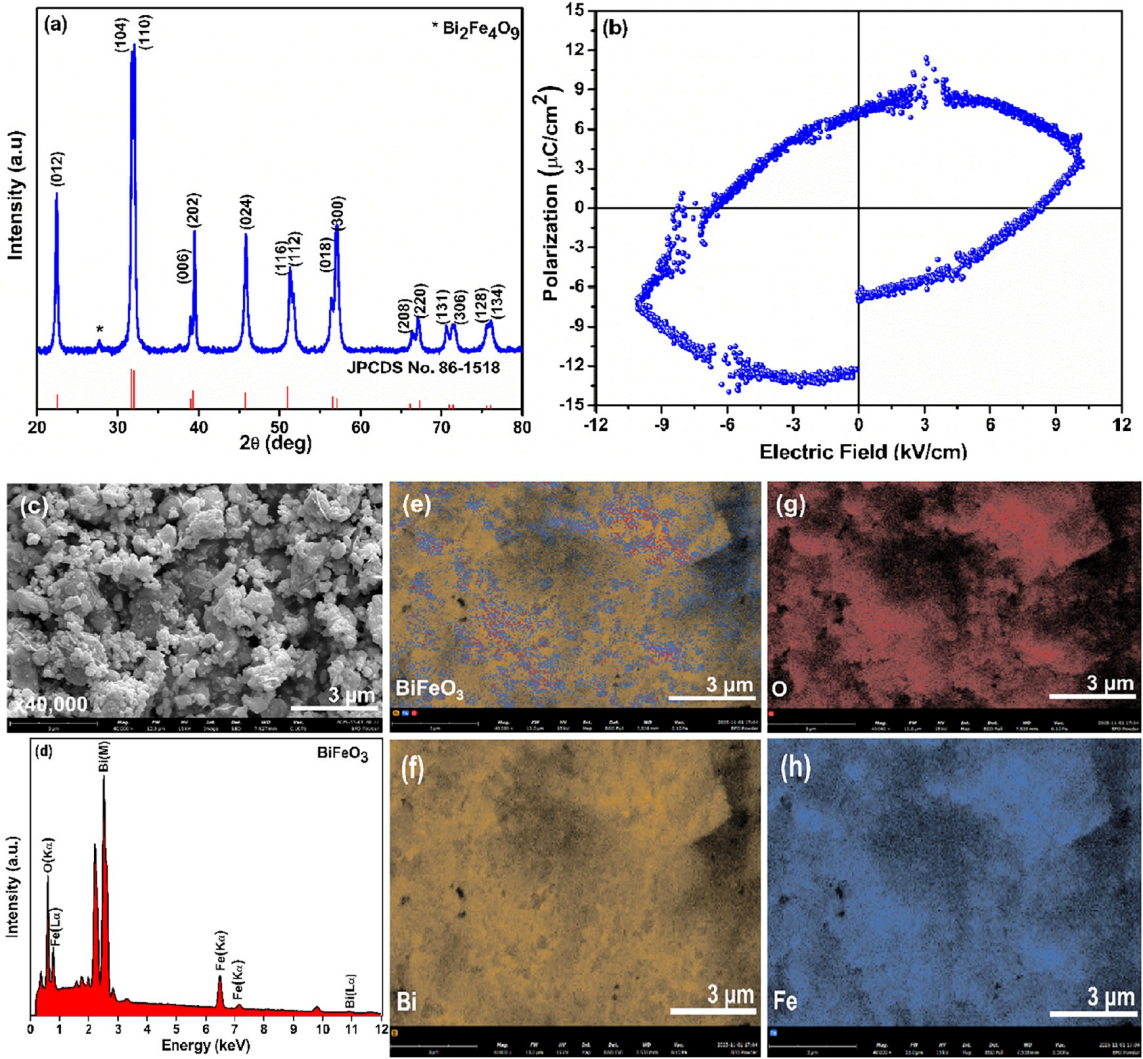
Characterization of BiFeO_3_ powders. (a) XRD pattern, (b) Ferroelectric hysteresis loops, (c) SEM image, (d) EDS spectra, and (e–h) EDS elemental mapping.

### 3.2 Structural and Physicochemical Properties of CA and CA-MFO Membranes Compared with CSs

The chemical groups of the CA and CA-MFO composite separators were analyzed by Fourier transform infrared spectroscopy, as shown in Figure 2. The spectral characteristic absorption band at ∼1750 cm^–1^ corresponds to the stretching vibration of the carbonyl group of C=O, while the bending vibration of the methyl group of C–CH_3_ is observed at ∼1366 cm^–1^. The C–O stretching band appears at ∼1234 cm^–1^, and the asymmetric stretching of C–O–C groups is detected at ∼1048 cm^–1^. These characteristic peaks confirm the presence of the cellulose acetate backbone as shown in Figure 2.^18,21,38,39^ The incorporation of oxide nanoparticles did not generate new peaks, indicating that oxides were physically embedded rather than chemically bonded to the polymer matrix. However, small shifts and variations in band intensity particularly near the carbonyl and C–O–C regions suggest interfacial interactions such as hydrogen bonding of CA and the CA-MFO. In contrast, the commercial separator exhibited simple absorption peaks at ∼2870, 1452, 1167, and 805 cm^–1^, corresponding to C–H stretching, CH_3_ bending, C–H bending, and C–C stretching vibrations of the polypropylene, respectively.^4,40^ The absence of polar functional groups such as C=O, C–O, and C–O–C demonstrates the intrinsically low polarity of CS, which directly correlates with its limited electrolyte wettability compared to CA-MFO separators.

**Figure 2:**
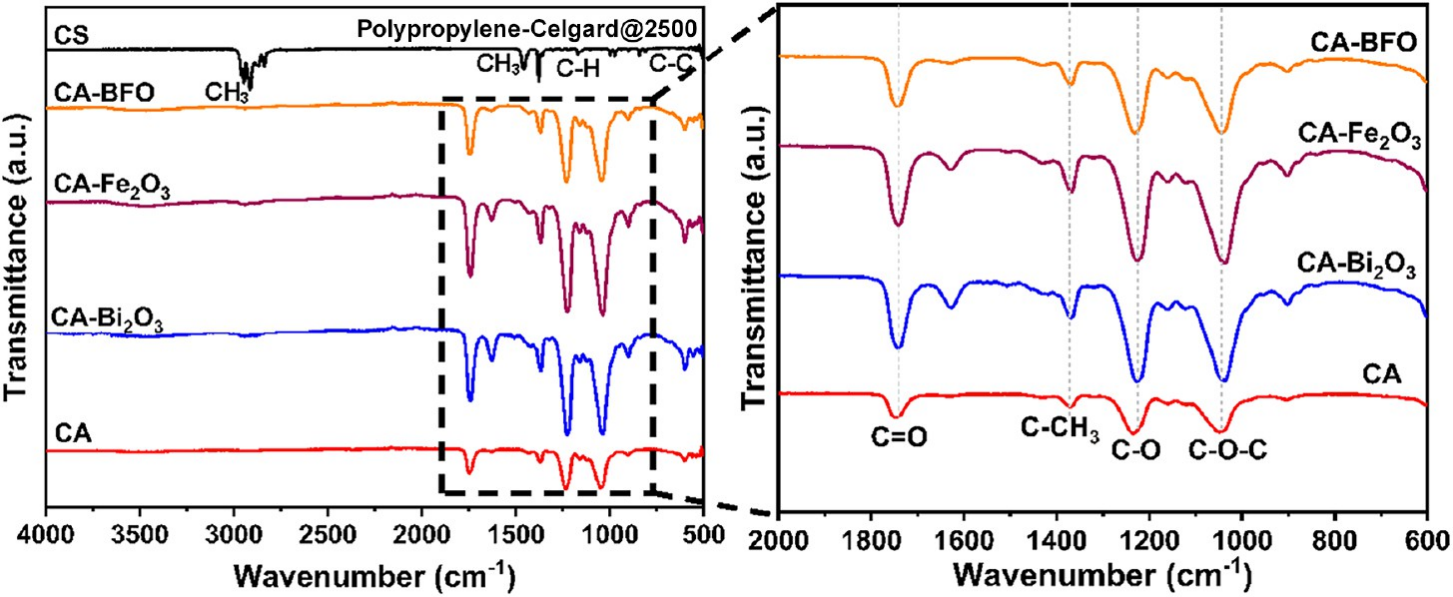
FT-IR spectra of cellulose acetate separators, composites with Bi_2_O_3_, Fe_2_O_3_, and BiFeO_3_, and the commercial separator.

The surface morphology of the electrospun CA and CA-MFO nanofiber separators was examined by using SEM, as shown in Figure 3. All electrospun separators exhibit a uniform, bead-free fibrous structure composed of randomly oriented nanofibers. The fibers are distributed homogeneously at the nanoscale, forming an interconnected porous network characteristic of well-optimized electrospinning conditions. Such morphology is highly desirable for LIBs. The SEM image of the CA-BFO nanofiber separators, shown in Figure 3g, reveals slightly thinner and more homogeneous fibers compared to those of the CA-Bi_2_O_3_ and CA-Fe_2_O_3_ separators shown in Figure 3c–f. This improvement suggests enhanced electrospinning stability, which can be attributed to the ferroelectric nature of the BFO nanoparticles incorporated into the CA matrix. In contrast, the CA membrane exhibits smoother, bead-free fibers, whereas the incorporation of multifunctional oxides results in a slightly rougher surface and occasional nanoparticle agglomerates embedded within the fiber matrix, indicating strong interfacial interactions between the oxide particles and polymer chains. Meanwhile, the CS separator displays a dense, nonporous surface, typical of polypropylene materials. This compact structure limits the electrolyte uptake and ionic mobility.

**Figure 3:**
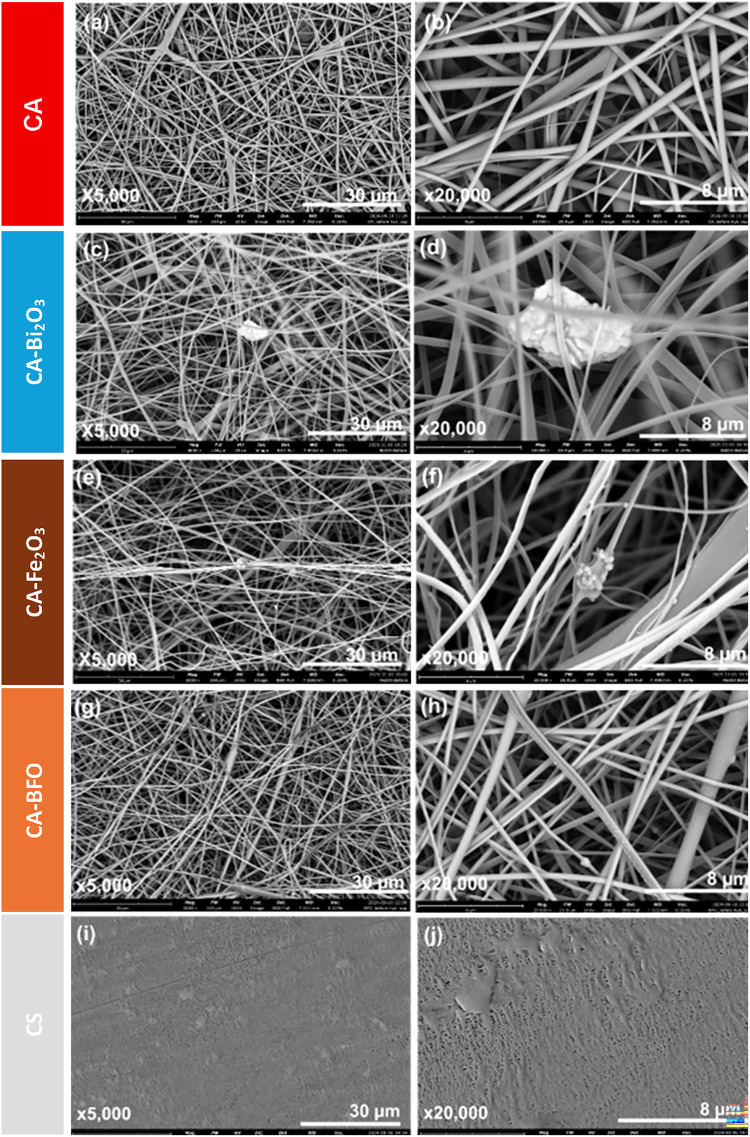
SEM images of separators: (a, b) CA; (c, d) CA-Bi_2_O_3_; (e, f) CA-Fe_2_O_3_; (g, h) CA-BFO, and (i, j) commercial separator at ×5000 and ×20,000 magnifications.

### 3.3 Regeneration Process of the Composite Separators

The regeneration process of the composite separators involves the chemical conversion of CA into regenerated cellulose (RE:C) through alkaline hydrolysis. This step is critical for improving the hydrophilicity, ionic transport, and mechanical flexibility of the electrospun membranes. During regeneration, the acetyl groups (COCH_3_) of CA are removed in alkaline medium 0.05 M KOH in aqueous ethanol, restoring hydroxyl (OH) functionalities along the cellulose backbone. The removal of these acetyl substituents enhances the affinity of the regenerated fibers toward the liquid electrolyte and promotes stronger hydrogen bonding networks between polymer chains, thereby improving separator stability and wettability. Scheme 3 illustrates the chemical mechanism, where the hydrolysis of CA leads to the formation of RE:C through the replacement of acetate groups with hydroxyl groups. The regeneration process preserves the fibrous morphology of the electrospun mats, maintaining their interconnected porosity, which is essential for efficient lithium-ion transport in electrochemistry.

**Scheme 3:**
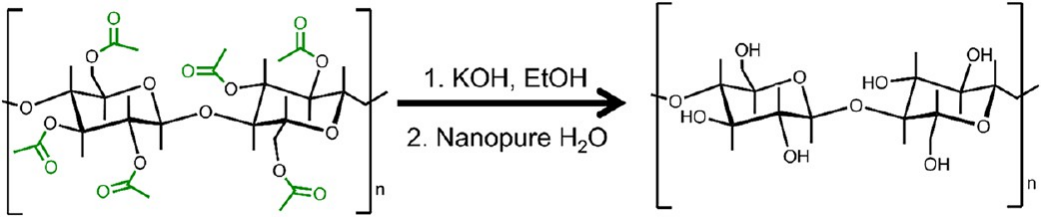
Chemical Mechanism of the Regeneration Process, Illustrating the Alkaline Hydrolysis of CA into RE:C through the Removal of Acetate Groups and Restoration of Hydroxyl Functionalities

FTIR spectroscopy was used to prove the composite formation of RE:C and multifunctional oxide separators RE:C-MFO after KOH in EtOH treatment, presented in Figure 4, together with the commercial separator in the wavenumber range from 500 to 4000 cm^–1^. All regenerated membranes exhibited the characteristic absorption bands of cellulose. The broad peak centered around ∼3450 cm^–1^ is attributed to the stretching vibration of the hydroxyl groups to OH, after alkaline treatment due to the hydrolysis process of CA groups.^41^ The peak observed at ∼1640 cm^–1^ is assigned to the C–CH_3_ bending vibrations of methyl groups.^42^ The incorporation of multifunctional oxides did not eliminate the characteristic CA peaks but induced subtle intensity changes, indicating weak interactions between the oxide particles and hydroxy-carbonyl functionalities. These results confirm that regenerated CA and CA-MFO membranes retain the polymeric backbone while exposing additional hydroxyl functionalities that may enhance electrolyte wettability compared to conventional separators.

**Figure 4:**
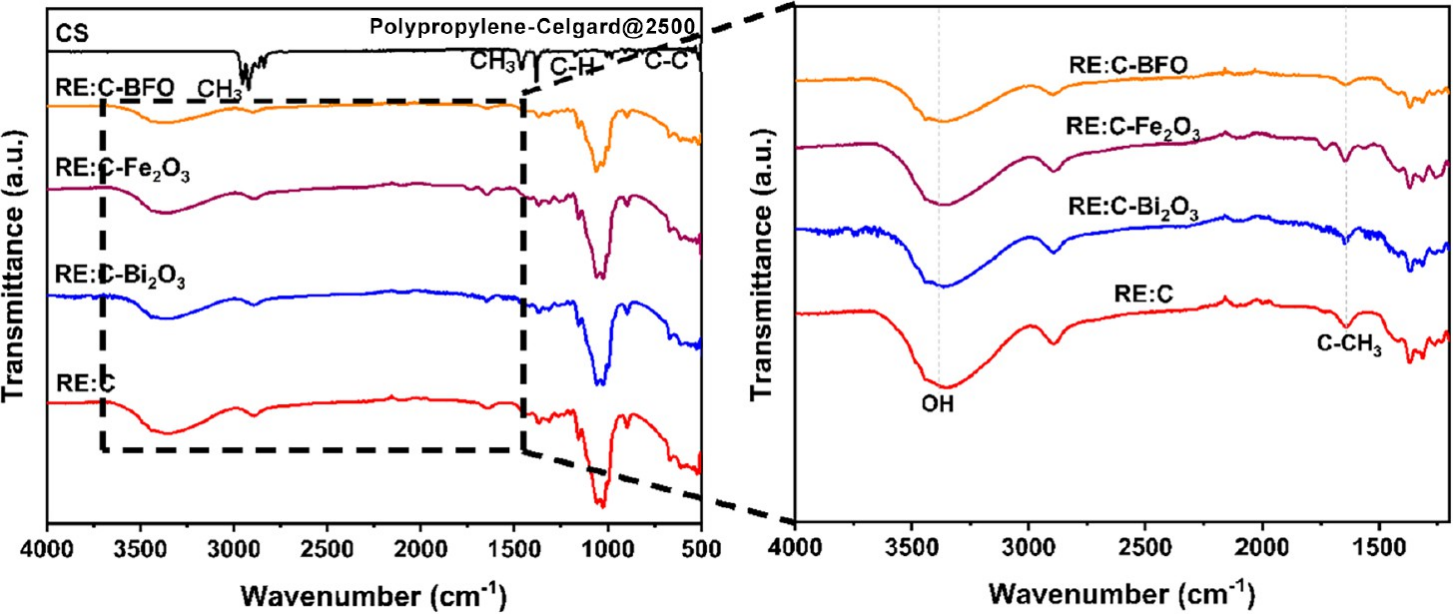
FT-IR spectra of regenerated CA and CA–MFO separators after KOH in EtOH treatment compared with the commercial separator (CS, untreated).

The surface morphology of the regenerated separators after the alkaline hydrolysis is shown in Figure 5. All RE:C and RE:C-MFO separators retained their fibrous structures after the regeneration process, confirming that the alkaline treatment did not damage the electrospun network. The fibers appear more uniform and slightly rougher than those of the pristine CA separator, indicating partial surface etching and exposure of hydroxyl groups formed during deacetylation. In particular, the RE:C-BFO separator exhibits thinner and more homogeneous fibers, likely resulting from enhanced electrospinning stability and stronger interfacial interactions between the ferroelectric BFO nanoparticles and the regenerated cellulose matrix, which are shown in Figure 5i,j. Morphological characterization of the separator was further evaluated in terms of fiber uniformity and porosity, as both parameters critically affect the electrochemical performance and safety of LIBs. It is well-established that defect-free, interconnected fibrous networks promote electrolyte absorption and facilitate efficient Li^+^ transport between electrodes, while simultaneously preventing internal short circuits due to improved mechanical strength. Moreover, an optimal porosity in the range of 40 to 60% is desirable to ensure a balance between ionic conductivity and mechanical robustness necessary for stable battery operation.^43^ Such a morphology, achieved in the RE:C-MFO membranes, is therefore highly beneficial for achieving superior electrolyte wettability and ion transport performance compared to the dense, nonporous polypropylene surface observed in the CS separator.

**Figure 5:**
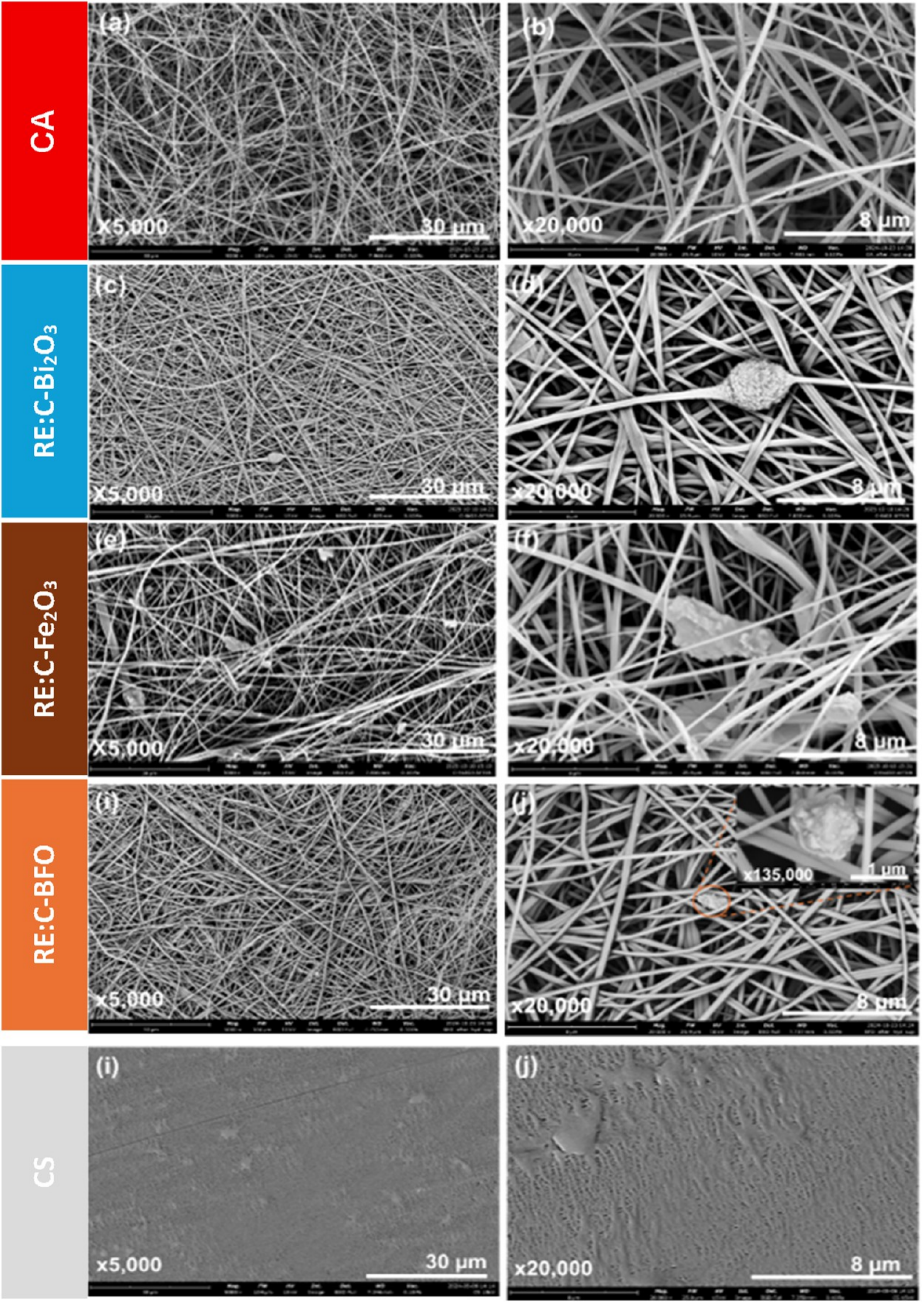
SEM images of regenerated separators: (a, b) RE:C; (c, d) RE:C–Bi_2_O_3_; (e, f) RE:C–Fe_2_O_3_; (g, h) RE:C-BFO, and (i, j) commercial separator at ×5000 and ×20,000 magnifications.

The thermal stability of the regenerated cellulose composite separators was evaluated by thermogravimetric analysis (TGA) under a nitrogen atmosphere and compared with the CS. The corresponding TGA and derivative thermogravimetric (DTG) curves are presented in Figure 6a–e. As shown in Figure 6a, the CS retained 95.7% of its mass prior to the principal degradation region, exhibiting an initial mass loss of only 2.36% and a final residual mass of 9.83%. Its DTG profile displayed a single and well-defined decomposition peak centered at approximately 270 °C, characteristic of the relatively homogeneous thermal degradation behavior of polypropylene-based materials. The low initial mass loss and narrow DTG peak indicate minimal volatile content and high compositional uniformity. In contrast, all regenerated cellulose-based composite separators shown in Figure 6b–e exhibited multistep decomposition profiles typical of chemically heterogeneous cellulose composite systems. Under inert N_2_ conditions, the residual mass observed at 600 °C is primarily associated with carbonaceous char formation and thermally stable inorganic oxide phases. The initial mass loss, ranging from 5.44 to 7.80%, is attributed to the evaporation of physically adsorbed moisture and residual volatile species associated with the regenerated cellulose matrix and oxide surface functionalities. The principal DTG decomposition peak for all regenerated separators appeared near 300 °C, corresponding to cellulose depolymerization and glycosidic bond cleavage. Notably, this degradation temperature shifted to slightly higher values compared with the CS separator, indicating that the incorporation of multifunctional oxide nanoparticles improves the thermal resistance and delays the onset of primary thermal degradation within the regenerated cellulose matrix. The flame resistance and thermal stability tests shown in Figure 6f–h demonstrate that RE:C-BFO resists shrinkage and maintains its morphology after 20 s of flame exposure, whereas CS melts due to its thermoplastic polypropylene nature.

**Figure 6:**
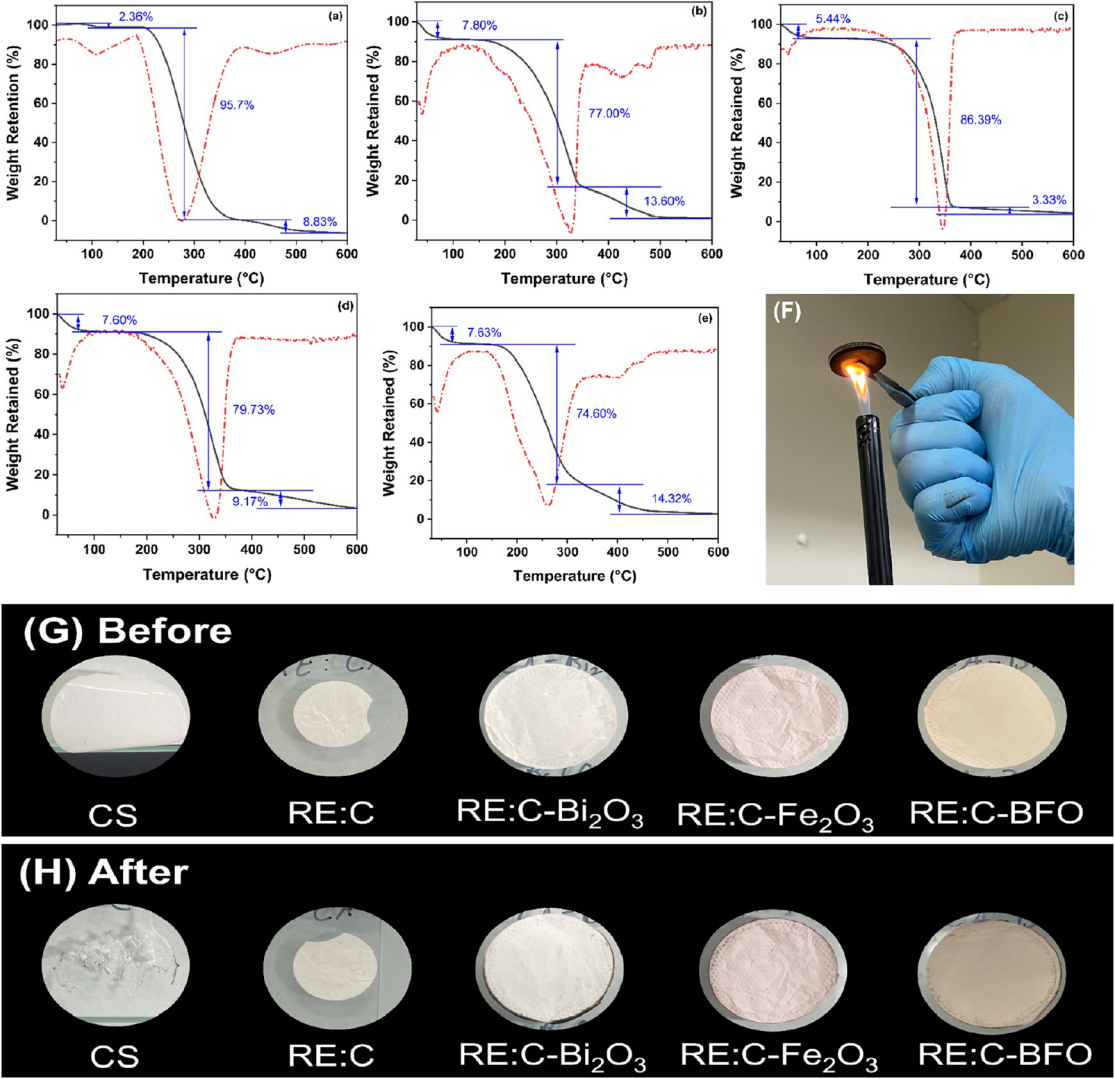
TGA and DTG curves of (a) CS, (b) RE:C, (c) RE:C–Bi_2_O_3_, (d) RE:C–Fe_2_O_3_, and (e) RE:C-BFO separators. Photographs and analyses illustrate the physical properties of the regenerated RE:C and RE:C-MFO separators: (f) flame resistance test of the steel/separator/steel configuration after 20 s of flame exposure and (g, h) photographs of the RE:C, RE:C-MFO, and CS separators before and after thermal treatment for 20 s.

Table 2 summarizes the physical properties of the regenerated RE:C and RE:C-MFO separators in comparison with the commercial CS separator. The data show that the RE:C-BFO separator exhibits outstanding mechanical performance, with a tensile strength of 11.5 MPa and Young’s modulus of 611.5 MPa, the highest among all RE:C and RE:C-MFO separators. In contrast, the CS separator displays a stiffer and more brittle response typical of polypropylene, reflecting its lower flexibility also aligned with its higher nominal porosity at ∼88%. The superior mechanical robustness of RE:C-BFO, combined with a moderate thickness of 40 μm, ensures mechanical stability and easy handling during cell assembly. These trends are visually confirmed in Figure 7a,b, where RE:C-BFO maintains structural integrity upon bending, whereas the CS deforms and shows limited elasticity. The electrospun separators were immersed in the liquid electrolyte for 24 h at room temperature to ensure full wetting and equilibrium absorption prior to measurement. The electrolyte uptake data in Table 2 show that the RE:C-BFO separator exhibits a high uptake of ∼382%, nearly twice that of the CS at ∼200%, confirming its affinity toward liquid electrolytes.

**Table 2:** Physical, Structural, and Mechanical Properties of Regenerated and CS Separators

separators	thickness [μm]	porosity [%]	electrolyte uptake [%]	pore size [μm]	tensile strength [MPa ]	Young’s modulus [MPa]
CS	25	∼88	∼200	0.04	16.6	528.2
RE:C	70	∼58	∼427	1.46	3.9	336.8
RE:C–Bi_2_O_3_	50	∼59	∼433	0.96	2.9	190.7
RE:C–Fe_2_O_3_	50	∼57	∼564	1.40	7.4	355.6
RE:C-BFO	40	∼63	∼382	0.91	11.5	611.5

**Figure 7:**
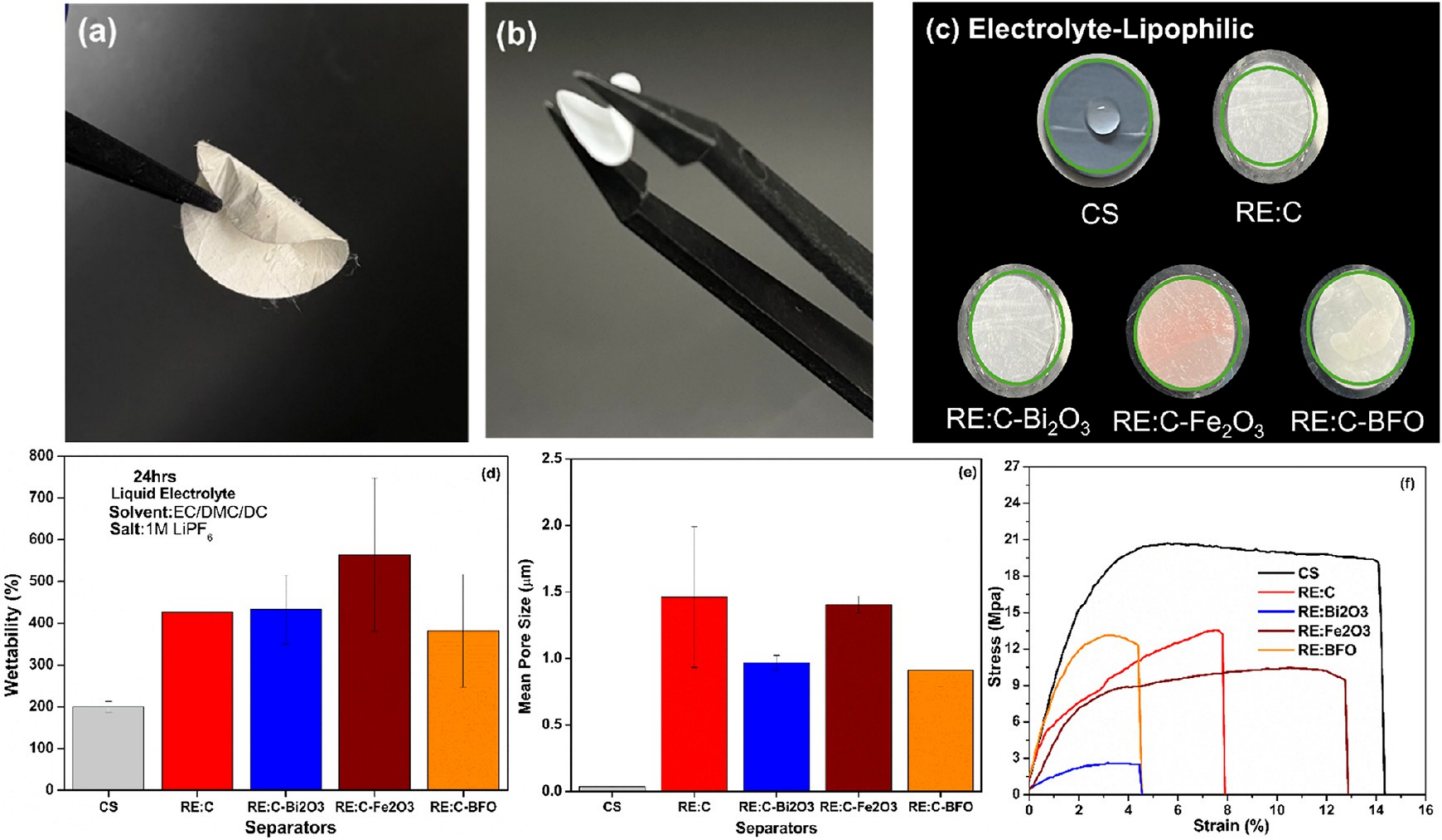
Photographs and analyses illustrate the physical properties of the regenerated RE:C and RE:C-MFO separators. (a) Flexibility of the RE:C-BFO separator compared to (b) CS separator. (c, d) Contact angle and wettability analysis illustrating the liquid electrolyte (1 M LiFP_6_ in EC/DMC/DEC) absorption behavior of the RE:C and RE:C-MFO separators compared to the CS separator. (e) Comparison of mean pore sizes for RE:C, RE:C-MFO, and CS separators. (f) Stress–strain curves illustrating the mechanical properties of the RE:C, RE:C-MFO, and CS separators.

This result is consistent with the wettability behavior observed in Figure 7c,d, where RE:C-BFO displays a pronounced *lipophilic* surface, evidencing strong compatibility with the organic electrolyte (1 M LiFP_6_ in EC/DMC/DEC). In contrast, the CS separator displays a weaker interaction with the electrolyte, leading to incomplete wetting and lower electrolyte retention. The enhanced wettability and ionic transport of RE:C-BFO are attributed to its interconnected pore network, characterized by an average pore size of 0.91 μm and porosity of ∼63%, which facilitate efficient electrolyte penetration while preserving mechanical integrity as shown in Table 2. In contrast, CS exhibits a higher nominal porosity at ∼88% and a fine microporous texture at 0.04 μm, which limits electrolyte infiltration and reduces ionic conductivity, as seen in Figure 7e. The stress–strain curves shown in Figure 6f further confirm that the RE:C-BFO separator achieves an optimal combination of ductility and tensile strength compared to the RE:C, RE:C–Bi_2_O_3_, and RE:C–Fe_2_O_3_ separators as shown in Table 2. This behavior reflects improved interfacial interactions between the BFO nanoparticles and the cellulose matrix, which enhance load transfer and mechanical integrity within the fibrous network. Overall, the RE:C-BFO separator achieves an optimal balance among flexibility, electrolyte wettability, mechanical integrity, and thermal safety, establishing it as a promising and sustainable alternative to conventional polypropylene separators for high-performance lithium-ion batteries.

### 3.4 Electrochemical Performance in Lithium-Ion Batteries

Figure 8 shows the electrochemical impedance analysis of the LIBs with graphite anodes and different separators before charge–discharge cycling. The spectra were fitted using the R­(CR)­(CRW) equivalent circuit model in FitMyEIS software, which enables separation of contribution from ohmic conduction, interfacial resistance, and charge transfer processes at the electrode/electrolyte interface. All batteries exhibited a characteristic semicircle in the high- to medium-frequency region, associated with *R*_SEI_, the resistance of the solid-state interface layer, which was formed due to the passivation reaction between the electrolyte and the electrode. CPE1 and CPE2 are two constant phase elements associated with interfacial resistance. *R*_ct_ is the charge transfer resistance^44^ followed by a linear Warburg tail at low frequencies, *W* is the Warburg impedance related to the diffusion of lithium ions Li^+^ within the electrode, and *R*_s_ is the solution resistance that corresponds to the combined resistance of the electrolyte, separator, and current collectors and is obtained from the high-frequency intercept of the *x*-axis.^24^ The first semicircle, governed by *R*_SEI_, reflects the ion conductivity and stability of the solid electrolyte interphase formed on the graphite anode, while the second semicircle represents the *R*_ct_ associated with the activation energy of Li^+^ intercalation at the graphite electrolyte interface. The low-frequency inclined tail is described by the Warburg diffusion element, defined as^45^

1 $$W=\sigma_{W}\omega^{-1/2}\left(1-j\right)$$

where σ_*W*_ is the Warburg coefficient and ω is the angular frequency. The lithium-ion diffusion coefficient can be obtained from the plots in the low-frequency region according to the equation^45^

2 $$D_{\mathrm{Li}}=\frac{R^{2}T^{2}}{2A^{2}n^{4}F^{4}C^{2}\sigma_{W}^{2}}$$

where *R* is the gas constant, *T* is the temperature (298 K), *n* is the number of electrons transferred (*n* = 1), A is the area of the electrode surface (1.327 cm^2^), *F* is Faraday’s constant, *C* is the lithium-ion concentration in the electrode (assumed as 1.0 × 10^–3^ mol cm^–3^), *D*_Li_ is the lithium-ion diffusion coefficient, and σ_*W*_ is the Warburg coefficient obtained from the linear fitting of *Z*’ versus ω^–1/2^ in the low-frequency region.

**Figure 8:**
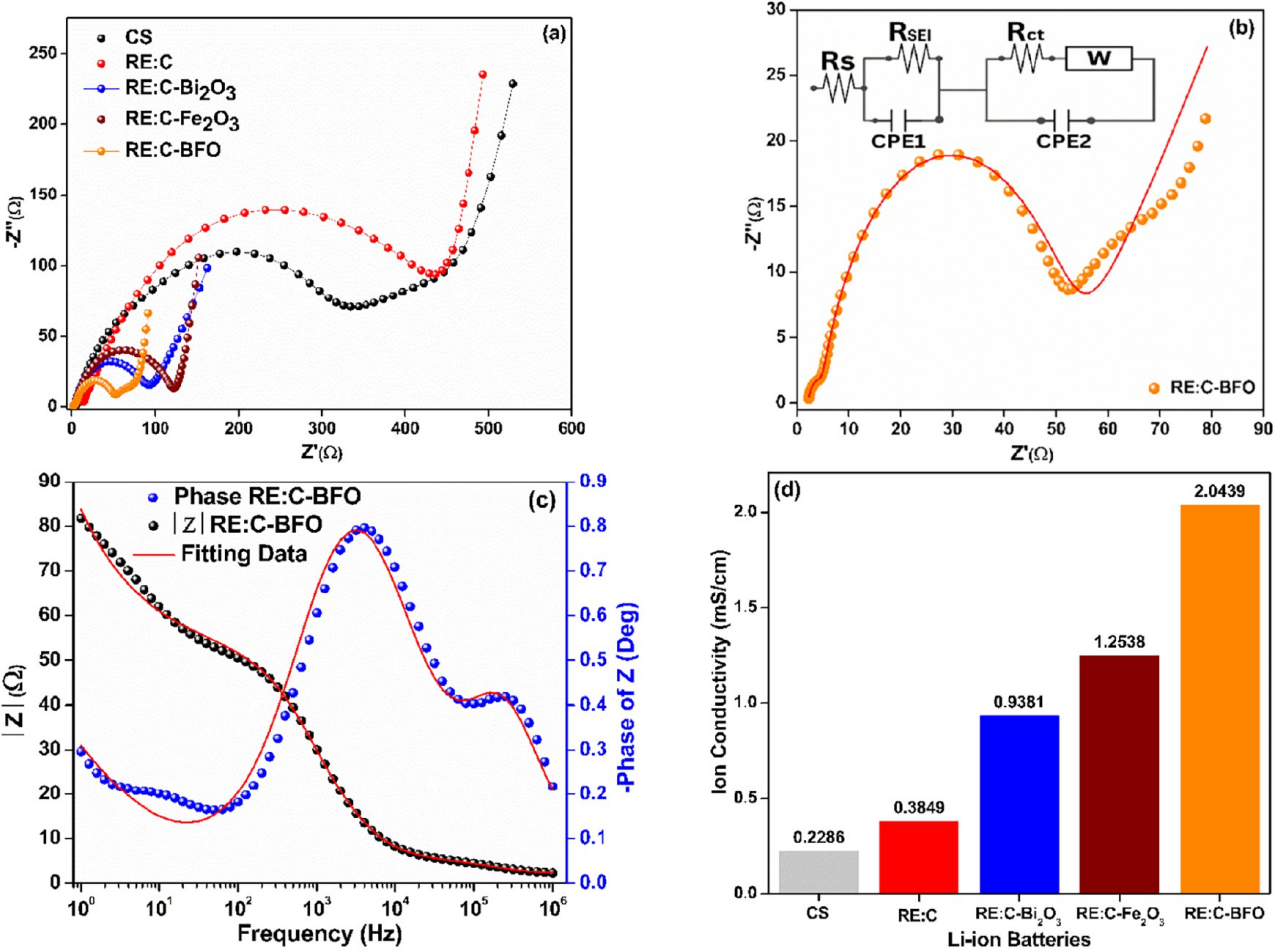
Electrochemical impedance spectroscopy analysis of all Li-ion cells using graphite anodes and different separators before charge–discharge cycling. (a) Nyquist plots for CS, RE:C, and RE:C-MFO separators; (b) Nyquist plot of the RE:C-BFO cell fitting using an equivalent circuit R­(CR)­(CRW) model (inset); (c) Bode plot (|*Z*| and phase) of the RE:C-BFO separator with the fitting curve; and (d) ionic conductivity for CS, RE:C, and RE:C-MFO.

Table 3 presents a comparison of the fitted impedance parameters, revealing a strong dependence of the electrochemical response on separator composition. The RE:C-BFO separator exhibited the lowest *R*_s_ value of 2.19 Ω, indicating efficient ion conduction through its interconnected regenerated cellulose network. Its *R*_SEI_ resistance remained extremely low at 2.24 Ω, noticeably smaller than that of the CS separator, which reached 33.82 Ω, and the pristine regenerated cellulose separator, which reached 49.99 Ω. This reduction demonstrates that BiFeO_3_ nanoparticles promote the formation of a more homogeneous and conductive *R*_SEI_ layer. Similarly, the charge transfer resistance followed the same tendency; RE:C-BFO presented a value of only 48 Ω, markedly lower than that of the CS separator at 300 Ω and the pristine regenerated cellulose separator at 298 Ω. The low-frequency behavior further confirms the enhanced Li^+^ mobility in the RE:C-BFO separator. The Warburg parameter decreased to 85.8 Ωs^–1/2^, enabling the highest *D*_Li_ in the system, calculated as 1.39 × 10^–13^ cm^2^/s, which is nearly 1 order of magnitude higher than that of the CS separator. This behavior reflects a more favorable pathway for ion transport within the separator/electrode interface. The reliability of the EIS fitting was assessed using the goodness-of-fit parameter χ^2^. As shown in Table 3, all fitted spectra exhibited low χ^2^ values in the range of 10^–7^ to 10^–6^, confirming that the selected R­(CR)­(CRW) equivalent circuit provides an accurate representation of the impedance response. For the calculation of the lithium-ion diffusion coefficient, an electrode area of 1.327 cm^2^ was used, and the Li^+^ concentration term corresponds to the lithium concentration in the graphite electrode. As shown in Figure 8d, this enhanced transport correlates directly with ionic conductivity that the RE:C-BFO separator achieved 2.0439 mS/cm, far exceeding the pristine RE:C separator at 0.3849 mS/cm and the CS at 0.2286 mS/cm. Overall, the combined reduction in *R*_s_, *R*_SEI_, and *R*_ct_, together with the substantial increase in ionic conductivity and diffusion coefficient, demonstrated that incorporating BiFeO_3_ nanoparticles into regenerated cellulose produces a highly efficient ion transport environment. These results position RE:C-BFO as the most electrochemically favorable separator in the system, providing a solid mechanistic foundation for its superior cycling performance in lithium-ion batteries.

**Table 3:** Comparison of Fitted EIS Parameters and Goodness-of-Fit Values for Li-Ion Batteries with Graphite Anodes Using the R­(CR)­(CRW) Equivalent Circuit Model and Different Separators

separators	*R*_s_ [Ω]	*R*_SEI_ [Ω]	CPE1 [F]	*R*_ct_ [Ω]	CPE2 [F]	*W* [Ωs^–1/2^]	*D*_Li_ [cm^2^/s]	χ^2^
CS	5.44	33.82	1.05 × 10^–5^	300	1.67 × 10^–3^	8.36 × 10^3^	1.85 × 10^–15^	1.92 × 10^–7^
RE:C	11.63	49.99	1.55 × 10^–3^	298	5.76 × 10^–4^	1.48 × 10^4^	7.47 × 10^–16^	6.95 × 10^–7^
RE:C–Bi_2_O_3_	3.18	1.59	1.26 × 10^–2^	76	1.42 × 10^–4^	192	2.82 × 10^–14^	1.02 × 10^–7^
RE:C–Fe_2_O_3_	3.57	1.59	1.25 × 10^–2^	102	1.71 × 10^–4^	170	3.59 × 10^–14^	2.48 × 10^–6^
RE:C-BiFeO_3_	2.19	2.24	1.58 × 10^–6^	48	6.39 × 10^–5^	85.8	1.39 × 10^–13^	9.65 × 10^–7^

Figure 9 presents the galvanostatic charge–discharge profiles and cycling performance of graphite anodes assembled with CS, RE:C, and RE:C-MFO separators under current densities of 0.2 mA/cm^2^ for the first five cycles and 0.5 mA/cm^2^ up to the 500th cycle (corresponding to C-rates of C/25 and C/10). In Figure 9a,b, the initial discharge capacity values of the CS for the first, second, 100th, and 500th cycles were 346, 286, 180, and 41 mAh/g, respectively. In Figure 9c,d, the discharge specific capacity values of the RE:C separator for the first, second, and fifth cycles were 51, 18, and 8 mAh/g at a current density of 0.2 mA/cm^2^, respectively. In Figure 9e,f, the discharge specific capacity values of the RE:C–Bi_2_O_3_ separator for the first, second, 100th, and 500th cycles were 360, 261, 131, and 42 mAh/g, respectively. In Figure 9g,h, the discharge specific capacity values of the RE:C–Fe_2_O_3_ separator for the first, second, 100th, and 500th cycles were 391, 265, 253, and 69 mAh/g, respectively. In Figure 9i,j, the discharge specific capacity values of the RE:C-BFO separator for the first, second, 100th, and 500th cycles were 326, 229, 252, and 72 mAh/g, respectively. For all batteries, the current density was 0.2 mA/cm^2^ for the first five cycles and 0.5 mA/cm^2^ for the remaining 500 cycles. The Coulomb efficiency values of the RE:C-MFO composite separators were 100%, as in Figure 9. The comparison of the specific capacity and retention for the LIBs is shown in Table 4. The batteries using the CS separator initially delivered a discharge capacity of 346.11 mAh/g, which decreased steadily to 180.51 mAh/g after 100 cycles, retaining 52% of its initial capacity. In contrast, the RE:C separator exhibited poor interfacial stability and limited Li-ion transport, achieving an initial capacity of only 51.99 mAh/g, which rapidly faded and prevented long-term cycling. Incorporating multifunctional metal oxide nanoparticles markedly improved electrochemical performance. The RE:C–Bi_2_O_3_ separator reached an initial capacity of 360.75 mAh/g, but its performance declined quickly, stabilizing at 131.73 mAh/g at the 100th cycle, corresponding to a capacity retention of 37%, indicating an insufficient SEI stabilization and higher long-term interfacial resistance. A more robust cycling response was obtained with the RE:C–Fe_2_O_3_ separator, which began with 391.91 mAh/g and maintained 253.09 mAh/g after the 100th cycle, achieving a retention of 65%, reflecting the beneficial redox activity and moderate polar character of Fe_2_O_3_.

**Figure 9:**
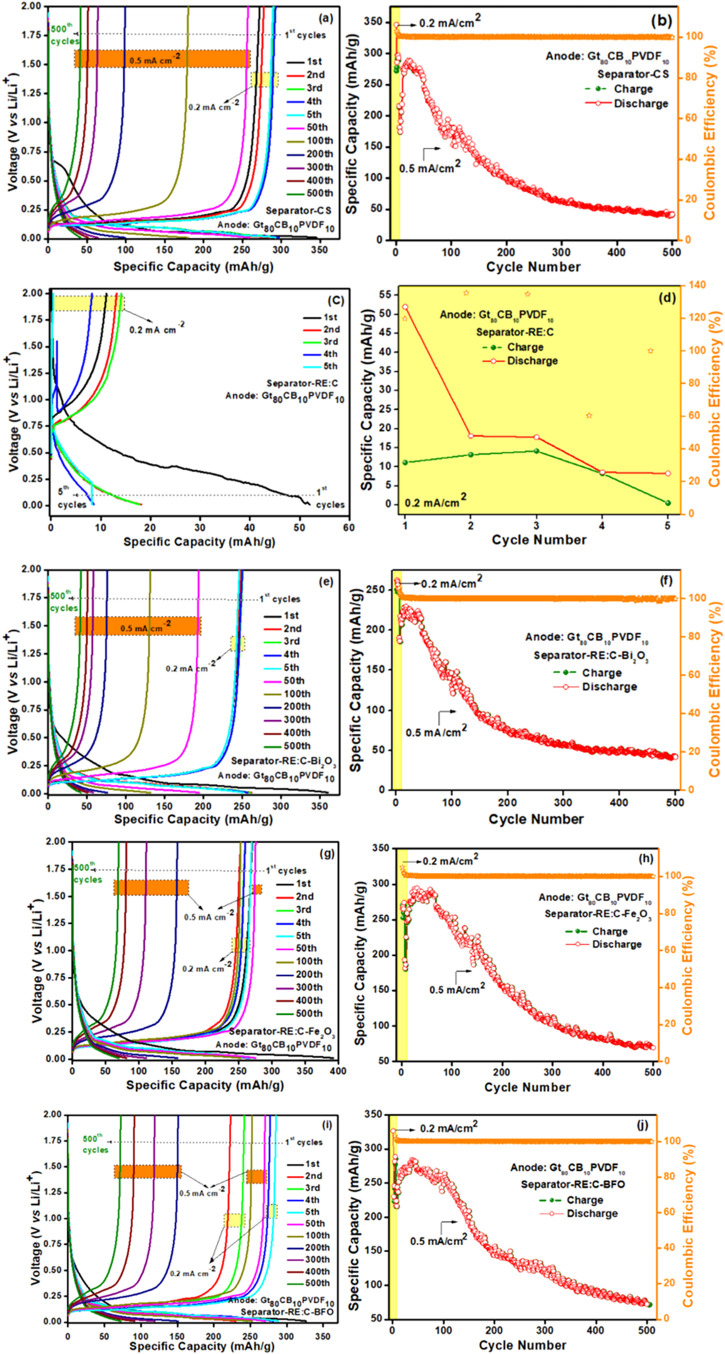
Charge–discharge profiles and specific capacity, coulombic efficiency in the function cycle number of all Li-ion cells using graphite anodes and different separators at various current densities from 0.2 to 0.5 mA/cm^2^, (a, b) CS separator; (c, d) RE:C separator; and (e, j) RE:C-MFO separators. All batteries were run over cycling at 0.5 mA/cm^2^ (first five cycles were run at 0.2 mA/cm^2^).

**Table 4:** Comparison of Specific Capacity between the 1st and 100th Discharge Cycles and Capacity Retention for the CS, RE:C, and RE:C-MFO Separators

separators	first specific capacity [mAh/g]	100th specific capacity [mAh/g]	capacity retention [%]
CS	346.11	180.51	52
RE:C	51.99		
RE:C–Bi_2_O_3_	360.75	131.73	37
RE:C–Fe_2_O_3_	391.91	253.09	65
RE:C-BFO	326.48	252.58	77

The best overall performance was observed for the RE:C-BFO separator, which delivered an initial capacity of 326.48 mAh/g and demonstrated excellent long-term stability by sustaining 252.58 mAh/g at the 100th cycle, resulting in the highest retention of 77% among all of the separators tested. This superior behavior is consistent with the improved ionic conductivity, reduced charge transfer resistance, and enhanced SEI formation evidenced in the EIS analysis, confirming that the ferroelectric and multiferroic properties of BiFeO_3_ facilitate more uniform Li^+^ flux, mitigate electrode polarization, and suppress interfacial degradation during cycling. Overall, the coupling of regenerated cellulose with BiFeO_3_ nanoparticles yields a separator capable of significantly enhancing capacity stability and cycling durability compared to those of both the pristine RE:C membrane and the CS separator.

## 4 Conclusions

The comprehensive characterization of the regenerated cellulose-based separators demonstrates that alkaline hydrolysis is an effective strategy for converting cellulose acetate into a hydrophilic, mechanically robust, and electrochemically active separator suitable for LIB applications. Structural and chemical analyses confirmed the complete removal of acetyl groups and restoration of hydroxyl functionalities, enabling the formation of stronger hydrogen bonding networks and significantly enhancing electrolyte affinity. SEM and pore structure evaluations revealed that the regenerated separators retained their interconnected nanofibrous morphology while exhibiting pore sizes and porosity levels that favored electrolyte uptake and ionic transport compared to the CS. Mechanical and thermal assessments further showed that regenerated separators, particularly those incorporating multifunctional oxides, exhibit superior flexibility, tensile stability, thermal tolerance, and flame resistance. Among all RE:C-MFO separators, the RE:C-BFO separator delivered the most balanced and superior performance, including high ionic conductivity, reduced *R*_s_, *R*_SEI_, and *R*_ct_, enhanced Li^+^ diffusion, and exceptional long-term cycling stability. These synergistic enhancements arise from strong interfacial interactions between BiFeO_3_ nanoparticles and the regenerated cellulose matrix, combined with the intrinsic ferroelectric behavior of BFO, which homogenizes the internal electric field, stabilizes the *R*_SEI_, and promotes uniform Li^+^ flux during cycling. Overall, the regeneration approach combined with multifunctional oxide reinforcement yields a new class of sustainable, high-performance separators that surpass CSs in electrochemical efficiency and safety, offering a promising platform for next-generation high-stability LIBs.
